# Real-world comparison of two molecular methods for detection of respiratory viruses

**DOI:** 10.1186/1743-422X-8-332

**Published:** 2011-06-29

**Authors:** S Asad Ali, James E Gern, Tina V Hartert, Kathryn M Edwards, Marie R Griffin, E Kathryn Miller, Tebeb Gebretsadik, Tressa Pappas, Wai- ming Lee, John V Williams

**Affiliations:** 1 Department of Pediatrics, Vanderbilt University School of Medicine, 1161 21st Avenue South, Nashville, TN, 37232, USA; 2 Department of Medicine, Vanderbilt University School of Medicine, 1161 21st Avenue South, Nashville, TN, 37232, USA; 3 Department of Preventive Medicine, Vanderbilt University School of Medicine, 1161 21st Avenue South, Nashville, TN, 37232, USA; 4 Department of Microbiology and Immunology, Vanderbilt University School of Medicine, 1161 21st Avenue South, Nashville, TN, 37232, USA; 5 Department of Pediatrics, University of Wisconsin School of Medicine and Public Health, 750 Highland Ave, Madison, WI, 53705, USA; 6 Department of Medicine, University of Wisconsin School of Medicine and Public Health, 750 Highland Ave, Madison, WI, 53705, USA

## Abstract

**Background:**

Molecular polymerase chain reaction (PCR) based assays are increasingly used to diagnose viral respiratory infections and conduct epidemiology studies. Molecular assays have generally been evaluated by comparing them to conventional direct fluorescent antibody (DFA) or viral culture techniques, with few published direct comparisons between molecular methods or between institutions. We sought to perform a real-world comparison of two molecular respiratory viral diagnostic methods between two experienced respiratory virus research laboratories.

**Methods:**

We tested nasal and throat swab specimens obtained from 225 infants with respiratory illness for 11 common respiratory viruses using both a multiplex assay (Respiratory MultiCode-PLx Assay [RMA]) and individual real-time RT-PCR (RT-rtPCR).

**Results:**

Both assays detected viruses in more than 70% of specimens, but there was discordance. The RMA assay detected significantly more human metapneumovirus (HMPV) and respiratory syncytial virus (RSV), while RT-rtPCR detected significantly more influenza A. We speculated that primer differences accounted for these discrepancies and redesigned the primers and probes for influenza A in the RMA assay, and for HMPV and RSV in the RT-rtPCR assay. The tests were then repeated and again compared. The new primers led to improved detection of HMPV and RSV by RT-rtPCR assay, but the RMA assay remained similar in terms of influenza detection.

**Conclusions:**

Given the absence of a gold standard, clinical and research laboratories should regularly correlate the results of molecular assays with other PCR based assays, other laboratories, and with standard virologic methods to ensure consistency and accuracy.

## Background

Respiratory viruses are common causes of human disease. Molecular detection techniques have allowed previously known viruses to be more reliably identified and new viruses to be discovered[1-10]. Molecular techniques such as real-time RT-PCR (RT-rtPCR) can be performed for each individual virus, or they can be combined into a multiplex RT-rtPCR assay. Other molecular methods that can target a single analyte or multiple viruses use a variety of nucleic acid detection based strategies, including microarrays[11], mass spectrometry [12], spectrally distinct bead microarrays[13], capillary electrophoresis [14], or microsphere flow cytometry[15]. The performance of molecular diagnostic methods, either RT-rtPCR or multiplex assays, typically has been evaluated by comparing them with conventional direct fluorescent antibody (DFA) or culture, with few direct comparisons reported between molecular assays. Moreover, there are few reports of laboratories collaboratively comparing methods between institutions. In this study, we compared a multiplex respiratory virus panel (Respiratory MultiCode Assay [RMA], EraGen Biosciences and U. Wisconsin-Madison) with RT-rtPCR (Vanderbilt University) for the detection of 11 common respiratory viruses.

## Methods

### Clinical specimens

Nasal and throat swabs were collected from children ≤ 12 months old who were admitted to the Monroe Carell Jr. Children's Hospital at Vanderbilt from September 1, 2005 through May 31, 2006 with acute respiratory illness, as part of the Tennessee Children's Respiratory Initiative [16]. All specimens collected during this period were included in the study. Within four hours of collection, the samples were divided into aliquots, lysis buffer added, and specimens were frozen at -80°C. Separate unthawed aliquots were sent to the research laboratories at Vanderbilt University and University of Wisconsin-Madison for RT-rtPCR and RMA testing, respectively.

### Real-time RT-PCR testing

Single RT-rtPCR experiments were performed for the following 11 viruses: respiratory syncytial virus (RSV), influenza A and B, human metapneumovirus (HMPV), human rhinovirus (HRV), parainfluenza 1, 2 and 3 (PIV1, PIV2, PIV3), and human coronaviruses Netherlands (NL-63), OC43, and 229E. RNA was extracted on an automated instrument (MagNApure Total Nucleic Acid extraction kit; Roche Applied Science), and refrozen at -80°C until each individual RT-rtPCR was performed. Primers and probes for HCoV-NL63, OC43, 229E, RSV, HMPV and HRV were obtained from the published literature[17-22]. Influenza primer and probe sequences were provided by Steve Lindstrom, Centers for Disease Control and Prevention [23]. Primers and probes for PIV1, 2 and 3 were designed using Primer Express version 2.0 (Applied Biosystems). Primer and probe sequences, concentration, and annealing temperatures are listed in Table 1. Twenty-five-μL reaction mixtures containing 5 μL of specimen RNA were tested using the QuantiTect Probe RT-PCR kit (QIAGEN) on a Smart Cycler II (Cepheid). Cycling conditions were 30 min at 50°C, 15 min at 94°C, and 45 cycles of 8 seconds at 94°C and 60 seconds at 55-66°C (Table 1). All RT-rtPCR assays were optimized and characterized using RNA runoff transcripts and were capable of detecting < 50 RNA transcript copies/reaction (data not shown). All samples were tested by a commercial RT-rtPCR assay for human beta-actin mRNA (Applied Biosystems) to ensure RNA integrity.

**Table 1:** Primer and probe sequences, reaction concentrations, and annealing temperatures used for RT-rtPCR assays.

Virus (target)	Forward primer	μM	Reverse primer	μM	Probe (5' to 3')	μM	°C	Ref
HRV (5' UTR)$	CY+AGCC+TGCGTGGC	0.8	GAAACACGGACACCCAAAGTA	0.8	TCCTCCGGCCCCTGAATGYGGC	0.1	60	[20]

1^st ^Round RSV (M)	GGAAACATACGTGAACAAGCTTCA	1	A: CATCGTCTTTTTCTAAGACATTGTATTGA	0.5	TGTGTATGTGGAGCCTTCGTGAAGCAAG	0.2	60	[19]
							
			B: TCATCATCTTTTTCTAGAACATTGTACTGA	0.5				

2^nd ^Round RSV (M)	GGCAAATATGGAAACATACGTGAA	1	TCTTTTTCTAGGACATTGTAYTGAACAG	1	CTGTGTATGTGGAGCCTTCGTGAAGCT	0.2	60	[18]

1^st ^Round HMPV (N)	CATATAAGCATGCTATATTAAAAGAGTCTC	1	CCTATTTCTGCAGCATATTTGTAATCAG	1	TGYAATGATGAGGGTGTCACTGCGGTTG	0.2	60	[21]

2^nd ^Round HMPV (N)	CATAYAARCATGCTATATTAAAAGAGTCTC	1	CCTATYTCWGCAGCATATTTGTAATCAG	1	CAACHGCAGTRACACCYTCATCATTRCA	0.2	60	*

HCoV OC43 (N)	CGATGAGGCTATTCCGACTAGGT	1	CCTTCCTGAGCCTTCAATATAGTAACC	1	TCCGCCTGGCACGGTACTCCCT	0.5	60	[22]

HCoV 229E (N)	CAGTCAAATGGGCTGATGCA	1	AAAGGGCTATAAAGAGAATAAGGTATTCT	1	CCCTGACGACCACGTTGTGGTTCA	0.5	60	

HCoV-NL (N)	AGGACCTTAAATTCAGACAACGTTCT	1	GATTACGTTTGCGATTACCAAGACT	1	TAACAGTTTTAGCACCTTCCTTAGCAACCCAAACA	0.5	66	[17]

PIV 1 (N)	GGATATCCTGCATGTCTCGG	1.5	GGCACTTTTAACGGTTCCATC	1.5	CCATAACAAGTAGTGCTGGTCTAAGAAAAGGATTCTTC	0.2	60	*

PIV 2 (N)	CACTTAAATATGGACTTGGAACAAGATG	1.5	TCCATCAGYTTTGGTGATTCTAATAGAG	1.5	CTACATTATCAGAGTCTAGGACCCATGGCCAA	0.2	60	*

PIV 3 (N)	AGCCATGCAACAGTATGTGACG	1.5	TTRGMTTCGTGTGTCACTCCAAGTTC	1.5	ATYTGAGCTTCRGCATCACGTGCTACTG	0.2	60	*

Flu A (M)	GACCRATCCTGTCACCTCTGAC	0.8	AGGGCATTYTGGACAAAKCGTCTA	0.8	TGCAGTCCTCGCTCACTGGGCACG	0.2	55	§

Flu B (NS)	TCCTCAACTCACTCTTCGAGCG	0.8	CGGTGCTCTTGACCAAATTGG	0.8	CCAATTCGAGCAGCTGAAACTGCGGTG	0.2	55	§

$ HRV forward primer: + = LNA base.

* this manuscript

§ CDC protocol (World Health Organization. CDC Protocol of Realtime RTPCR for Swine Influenza A (H1N1). April 28, 2009. http://www.who.int/csr/resources/publications/swineflu/CDCrealtimeRTPCRprotocol_20090428.pdf).

### RMA

RMA is a multitarget, high throughput technology that integrates multiplex PCR and microsphere flow cytometry [15,24,25]. While the Luminex technology can detect up to 80 targets in one reaction, this version of the assay included 19 viral targets. We report results for the same 11 viruses tested by the RT-rtPCR assays. Nucleic acid was extracted with Trizol reagents. The RMA assay was performed with primers and conditions as previously described, including the addition of an external insect virus spiked into each specimen to control for RNA extraction, RT, and PCR [15].

### Retesting with RT-rtPCR and RMA

Both sites were blinded to the results from the other laboratory until all data were analyzed. In order to address potential weaknesses indicated by discordant results between the two assays in the first round of comparison, both RT-rtPCR and RMA testing were modified for a second round of testing. The first round primers and probes for RSV and HMPV [19,21] were changed for the RT-rtPCR assay[18] and (Klemenc et al, unpublished data) and all samples were retested for these two viruses. In the RMA assay, the primers and probe for influenza A were modified and the cDNA synthesis protocol was changed from Promega reagents to the Applied Biosystems High-Capacity cDNA Kit.

### Statistical Analysis

Demographic and clinical variables of the study sample are described as frequencies and percent or median and interquartile ranges [IQR] as appropriate. The RMA and RT-rtPCR assays were compared using the test for inter-rater agreement (kappa coefficient). Kappa coefficient was calculated for any comparison in which the value of all data cells was ≥ 3, with a kappa ≥ 0.75 considered to indicate good agreement. Bootstrapping was used to estimate 95% confidence limits of kappa. Cycle threshold (Ct) numbers for influenza and RSV were compared between groups of specimens using the t-test.

## Results

A total of 225 infants hospitalized with respiratory symptoms were enrolled from September 2005 through May 2006. Samples from 222 children were available for study in both sites; three specimens were only available for RMA testing. Study children were 57% male with a median age of 13 weeks [IQR 7-33 weeks]. The admitting diagnoses of these infants were bronchiolitis (58%), upper respiratory infection (33%), and other (9%).

Rates of viral detection were compared using both assay methods. Restricting the analysis to the 11 viruses tested with both methods, at least one virus was detected in 174 (78.3%) samples by RMA and in 163 (73.4%) samples by RT-rtPCR. A total of 194 and 196 viruses were identified in these samples by RMA and RT-rtPCR, respectively (Table 2).

**Table 2:** Comparison of RMA and RT-rtPCR assays.

RMA (1)*					
**Virus**	RT-rtPCR (1)**	**Positive**	**Negative**	**Total**	**Kappa (95%CI)†**

RSV	Positive	70	8		
	Negative	28	116	222	0.66 (0.56-0.76)
					
HRV	Positive	50	13		
	Negative	6	153	222	0.78 (0.68-0.87)
					
HMPV	Positive	2	0		
	Negative	6	214	222	
					
Influenza A	Positive	4	21		
	Negative	3	194	222	
					
PIV 1	Positive	9	4		
	Negative	1	208	222	
					
PIV 2	Positive	1	0		
	Negative	0	221	222	
					
CoV NL63	Positive	5	1		
	Negative	3	211	220	
					
CoV OC43	Positive	4	1		
	Negative	0	215	220	
	**RT-rtPCR (2)**				
RSV	Positive	95	12		0.86 (0.78-0.92)
	Negative	3	110	220	
					
HMPV	Positive	4	1		
	Negative	2	211	218	
**RMA (2)**					
	**RT-rtPCR (1)**	Positive	Negative		
Influenza A	Positive	5	20		
	Negative	0	196	222	

*Kappa for agreement assessments is reported when all cells are ≥ 3.

RSV = respiratory syncytial virus, HRV = human rhinovirus, HMPV = human metapneumovirus, PIV = parainfluenza virus, CoV = coronavirus.

RMA (1) = first RMA run, RMA (2) = second RMA run with revised primers/probes, RT-rtPCR (1) = first RT-rtPCR run, RT-rtPCR (2) = second RT-rtPCR run with revised primers/probes.

### RSV

In the first round of testing, RMA detected more RSV (n = 98, 44%) than RT-rtPCR (n = 78, 35%). The kappa coefficient between the two tests was 0.66. RMA also differentiated between RSV sub-types A and B. Of the RSV positive samples detected by RMA, 67 were RSV A and 32 were RSV B (one sample had both RSV A and RSV B). Of the 67 RSV A identified through RMA, RT-rtPCR detected RSV in 65 (97%) of them. However, of the 42 RSV B detected by RMA, RT-rtPCR only detected RSV B in 6 (14.2%) samples.

In the second round, the primers and probes for the RSV RT-rtPCR assay were redesigned based on further analysis of published viral sequences [18]. Using these new primers and probes, RSV was identified in 107 (49%) samples with RT-rtPCR. The inter rater agreement for RSV between the repeat RT-rtPCR and the first RMA was 0.86. Of the 67 RSV A and 32 RSV B detected by the RMA assay, the repeat sample analysis detected RSV in 66 and 28 respectively. 12 samples were positive for RSV with repeat RT-rtPCR testing but negative for RSV with RMA testing; the mean Ct for these specimens was higher than the mean Ct for specimens detected by both assays (34.54 ± 3.18 vs. 28.58 ± 3.71, p < 0.001).

### Influenza

In the first round, the RT-rtPCR detected influenza A in 25 (11.2%) samples while the RMA detected influenza A in 7 (3.1%) samples. RT-rtPCR detected influenza B in 1 sample while RMA detected influenza B in a different sample. In the second round of testing, the influenza A RMA primers and probes were redesigned. Compared to 25 samples positive for influenza A by rRT-PCR, the repeat RMA assay detected 5 positive samples; all 5 were also detected by the RT-rtPCR assay. The mean Ct of the 5 influenza A specimens detected by the RMA assay was lower than the mean Ct of all influenza A specimens (30.02 ± 1.98 vs. 34.99 ± 1.88, p < 0.001).

### Human rhinovirus

RT-rtPCR detected HRV in 63 (28.2%) samples as compared to the RMA, which detected HRV in 56 (25.1%) samples (kappa coefficient 0.78). In two of the discordant samples, RT-rtPCR detected HRV while the RMA detected enterovirus.

### Human metapneumovirus

During the first round of testing, RMA detected HMPV in 8 (3.5%) samples while RT-rtPCR detected HMPV in 2 (0.9%) samples. In the second round using redesigned RT-rtPCR HMPV primers and probes, HMPV was detected in 7 samples, 5 of which were concordant with the results of the first RMA assay. The first RMA assay detected HMPV in 2 additional samples that were not detected by the repeat RT-rtPCR assay, while one sample positive for HMPV in the first RMA was negative in the second set of tests.

### Human coronaviruses

NL63 was detected in 8 (3.5%) samples through RMA and in 6 (2.7%) samples through RT-rtPCR. HCoV OC43 was detected in 5 (2.2%) samples through RMA and in 5 (2.3%) samples through RT-rtPCR. HCoV 229E was not detected in any sample by either assay.

### Parainfluenza viruses

PIV1 was detected in 10 (4.4%) samples through RMA and in 13 (5.8%) samples through RT-rtPCR. PIV 2 was detected in 1 (0.5%) sample through RMA and in 1 (0.5%) sample through RT-rtPCR. No PIV3 was detected by either method.

### Test/retest of respiratory viruses using the RMA assay

The comparison of the first and second run of RMA assay is summarized in Table 2. The most notable discrepancies between runs were for HRV and RSV.

## Discussion

The sensitivity and specificity of multiplex viral diagnostic assays have generally been compared to viral culture and DFA[1], instead of more sensitive RT-rtPCR methods [2,26-28]. Comparison of molecular methods with other sensitive molecular techniques, such as RMA with RT-rtPCR, is appropriate to determine whether the sensitivity for each analyte is sufficient for clinical or research use. Although both assays performed well for selected viruses in this study, the first round of comparison revealed differences in the rates of detection for specific viruses (Table 2). The RMA assay detected significantly more HMPV and RSV, while the RT-rtPCR assay detected more influenza A and HRV. Redesigning primers led to substantial improvements in the detection of HMPV and RSV by rRT-PCR, while there was little improvement in the performance of RMA for influenza virus with the change in primers. This discordance could have been due to sequence variation; there was insufficient residual specimen to sequence the viruses. However, both influenza A assays targeted regions of the matrix gene that are highly conserved among GenBank sequences (data not shown). The mean Ct of the influenza and RSV specimens detected only by the RT-rtPCR assay was significantly higher than those detected by the RMA assay, suggesting that the discordance may have been due to the sensitivity of the two assays for specimens with a lower viral load.

One recent study compared a commercial version of the RMA known as PLx-Respiratory Viral Assay (PLx-RVP) to RT-rtPCR and found good concordance, but only tested a portion of the PLx-RVP negative samples [29]. The RMA and PLx-RVP differ both in primer sequences, and in the method used to extract RNA and perform reverse transcription. Both of these factors are important determinants of assay sensitivity. Given that RNA viruses are prone to mutation[30], currently circulating field strains of these viruses should be regularly monitored for sequence divergences that would affect primers and probes used in PCR-based assays. This is particularly true for influenza, which undergoes considerable variation every year. Molecular assays are often targeted to conserved internal genes, but this is based on known sequences. Further, due to segment reassortment and antigenic shift, new internal gene segments may arise among influenza viruses infecting humans. For multiplex assays such as the RMA, inclusion of two probe sets that target two different parts of the viral genome might eliminate the risk that a single mutation would lead to nondetection.

Our study has some limitations. First, we did not use identical extraction methods and primers in the RT-rtPCR and RMA assays, and therefore discrepant results could have been due to differences in these processes or primer design. However, this was an intentional choice, since the specific goal of this study was to compare two "real-world" methods. Both laboratories have published extensively in the area of respiratory virus diagnostics and epidemiology, and have evaluated their independent methods. This study shows that collaboration between groups offers a valuable tool to assess assay performance impartially. Second, we did not use a gold standard test to confirm presence or absence of virus in cases of discrepancy between the two assays. We explored the option of sequencing of the discordant samples to detect sequence differences or false positives, but there was not enough residual specimen to allow this. Nonetheless, we repeated the testing of viruses that had major discrepancies with the assumption that if the repeats were positive, it provided reassurance that the positive samples were true positives. Third, the RT-rtPCR was only repeated for HMPV and RSV due to limited sample; there could have been discordances for other viruses. Fourth, the RMA assay was repeated with altered influenza primers while other primers were unchanged; it is possible that a change in influenza primers had an impact on the overall performance of the assay. One important consideration for any multiplex assay is the need to re-optimize for all targets when a change is made in any of the primers used. Finally, the results were not evaluated by DFA or culture, though the objective was to directly compare sequence-based methods.

In summary, for most viruses tested, there was good agreement between the two assays. However, discrepancies highlight the need for similar comparisons between molecular assays on a routine basis, so that weaknesses can be promptly identified and corrected. While investigators may be reluctant to make comparisons that might suggest deficiencies of particular methods, collaborative studies such as ours are critical to advance these diagnostic methodologies. Two other recent studies comparing molecular methods identified similar discrepancies [29,31]. In addition to clinical specimens validated by culture, respiratory virus proficiency panels distributed by quality control programs like the European Quality Control for Molecular Diagnostics (QCMD) [32], CDC, or College of American Pathologists, or commercial proficiency panels (e.g., ZeptoMetrix NATtrol™ Respiratory Validation Panels) provide an external quality control. Clearly, the overall comparison between molecular methods should consider the performance of each individual analyte. The goal of this study was not to determine which is the "best" assay; the choice of assay for a given purpose depends on many factors, including cost, available equipment and expertise, sample volume, and others. Multiplex assays may offer substantial savings in time and cost due to multiple analytes, and are generally preferred for clinical testing. Comparison of molecular diagnostic methods to culture and DFA is useful to establish validity, and ensure that cultivable viruses are correctly identified by sequence-based technology. However, our data show that molecular diagnostic methods also must be compared to other molecular techniques to assess performance, particularly because of the superior sensitivity of the molecular methods compared to culture and DFA.

## Competing interests

James Gern is on the scientific advisory board for and has stock options for 3 V Biosciences, has consulted for and has stock options for EraGen Biosciences, has consulted for Synairgen and Centocor, and has received research support from AstraZeneca and Merck. Kathryn M. Edwards receives research funding from sanofi-pasteur, Wyeth, Novartis, and CSL. Marie R Griffin has received grant support from MedImmune and Wyeth. John V. Williams has served as a consultant for MedImmune, Novartis and serves on the scientific advisory board of Quidel.

## Authors' contributions

AA and EKM performed RT-rtPCR and drafted the manuscript. TP and WML performed RMA testing. AA and TG performed the statistical analysis. AA, JEG, TVH, KME, MRG, and JVW conceived of the study, participated in its design and coordination, and helped to draft the manuscript. All authors read and approved the final manuscript.
